# Bridging classical wisdom and modern pedagogy: pre-service teachers’ cognitive engagement with Kutadgu Bilig

**DOI:** 10.3389/fpsyg.2026.1878026

**Published:** 2026-07-01

**Authors:** Kürşad Çağrı Bozkırlı, Emre Dağaşan

**Affiliations:** Department of Turkish and Social Sciences Education, Dede Korkut Faculty of Education, Kafkas University, Kars, Türkiye

**Keywords:** Kutadgu Bilig, pedagogical content knowledge, pedagogical interpretation, revised bloom’s taxonomy, teacher education

## Abstract

This study examines how pre-service Turkish language teachers interpret the pedagogical dimensions of Kutadgu Bilig and associate selected couplets with contemporary educational concepts. Employing a qualitative case study design, data were collected from 20 senior pre-service teachers through a structured mapping task involving 15 couplets. Participants were asked to associate each couplet with an educational concept and justify their choices. Data were analyzed via deductive thematic analysis using the Revised Bloom’s Taxonomy and Shulman’s Pedagogical Content Knowledge (PCK) frameworks as interpretive lenses. The findings indicate that while participants established pedagogical associations, they experienced varying degrees of difficulty in accurately relating them to intended concepts. The results suggest challenges in moving beyond concept recognition toward deeper pedagogical transfer. Participants’ justifications revealed significant differences in connecting culturally embedded messages with contemporary pedagogical frameworks. These findings highlight the need within teacher education programs for interpreting and applying pedagogical knowledge across diverse contexts. The study positions Kutadgu Bilig as a pedagogical resource for exploring the cognitive-pedagogical engagement of pre-service teachers.

## 1 Introduction

At the center of the rapid consumption habit in other words, the “consumption frenzy” imposed on humanity by the “modern world,” information now takes precedence. Like everything else, information is produced, gains meaning, and loses its significance extremely rapidly. Therefore, in such a period, the ability to process, interpret, and produce new knowledge by utilizing information has become as vital as fast access to accurate information. Adapting to this reality is significantly possible through education. This is because “education is to social life what nutrition and reproduction are to physiological life” (Dewey, 1996 as cited in Erdem and Demirel, 2002, p. 81). In other words, education is a vital necessity, and it is of great importance that this need aligns with societal expectations and the requirements of the age. In this context, contemporary scholarship suggests that education must go beyond mere information delivery to foster “culturally sustaining pedagogies” (Paris and Alim, 2017), which empower learners to navigate the tensions between tradition and the rapid shifts of the modern era. In this era, characterized by rapid development and change, education systems bear significant responsibilities for equipping younger generations with the knowledge and skills required by the 21st century (İlğan, 2021). To fulfil these responsibilities effectively, it is a necessity for education systems to possess contemporary characteristics that integrate historical wisdom with futuristic competencies.

Throughout history, every society has established education systems in line with its own needs and cultural values, and these systems have evolved and diversified over time until the present day (Şenkaloğlu, 2021). Like all other societies, Turkish society has possessed various education systems throughout its history. When the Turkish education system is mentioned, frequent and radical changes often come to mind. One of these changes was the reorganization of curricula according to the constructivist approach in 2006. According to this approach, students must participate in the learning process both mentally and physically. The ability of students to operate mental processes and, in particular, to inquire is of paramount importance for this approach (Arslan, 2007; Uymaz and Çalışkan, 2019). Curricula were revised again in 2019, and most recently in 2024. These changes aimed to ensure the use of higher order thinking skills and to achieve permanent learning that is compatible with daily life (Ministry of National Education, 2019). In this context, it can be stated that the expected outcome of the changes in both 2006 and 2019 was to raise individuals who play an active role in accessing information, question the information they reach, and can produce by utilizing this information in their lives. However, as Shulman (1987) emphasizes, the success of such curriculum reforms depends heavily on the teacher’s ability to transform theoretical content into meaningful pedagogical experiences. Within the scope of the “Turkey Century Education Model” introduced in 2024, it was expressed that the curricula just like previous programs aim to provide students with scientific practices such as research, inquiry, and observation, as well as an interdisciplinary perspective, in learning environments where active participation is offered (Ministry of National Education, 2024). This interdisciplinary outlook necessitates a profound synergy between historical cultural capital and contemporary pedagogical content knowledge.

Although the empirical context of this study is situated in Turkish teacher education, the problem it addresses is not context-bound. International research consistently reports persistent difficulties in enabling pre-service teachers to transform theoretical pedagogical knowledge into functional interpretive competence (Shulman, 1987; Renkl et al., 1996; Darling-Hammond, 2006). In this regard, the present study contributes to broader discussions on pedagogical content knowledge, inert knowledge, and culturally grounded sense-making by illustrating how these challenges manifest within teacher education systems across diverse educational contexts.

Among various approaches that classify cognitive development levels, Bloom’s Taxonomy is the most well-known (Akbulut Taş and Karabay, 2019; Altıparmak and Palabıyık, 2019). Bloom’s Taxonomy is a significant classification system with proven validity and reliability, designed to categorize students’ cognitive competencies and explain levels of cognitive knowledge (Yaşar and Kılıç, 2024). As noted by Gallagher (2020), cognitive interaction with complex instructional materials requires more than mere recognition; it necessitates an active “sense-making” process where the learner maps theoretical data onto practical frameworks. According to the classification originally made by Bloom et al. (1956), the stages of the cognitive domain comprising “knowledge, comprehension, application, analysis, synthesis, and evaluation” have undergone various changes over time. They were ultimately reorganized as remembering, understanding, applying, analysing, evaluating, and creating (Durukan and Demir, 2017; Yolcu, 2019). The theoretical synthesis of the Revised Bloom’s Taxonomy and Shulman’s Pedagogical Content Knowledge (PCK) framework provides a comprehensive diagnostic lens for this study; while PCK delineates the domain of knowledge required to transform classical literature into teachable content, the Revised Bloom’s Taxonomy measures the cognitive depth and complexity at which this knowledge is processed. There is a hierarchical connection between these levels (Oçak and Uzel, 2024). The prerequisite for progressing to a higher cognitive level is the successful completion of the lower level or levels. In this context, the taxonomy functions not just as a scale, but as a roadmap for developing high order thinking skills. According to the Revised Bloom’s Taxonomy, the cognitive process operates as follows:

Remembering: Retrieving relevant knowledge from long-term memory, recognizing, and recalling. Understanding: Constructing meaning from oral, written, and graphic messages through interpreting, exemplifying, classifying, summarizing, inferring, comparing, and explaining. Applying: Carrying out or using a procedure through executing or implementing. Analysing: Breaking material into constituent parts and detecting how the parts relate to one another and to an overall structure or purpose. Evaluating: Making judgments based on criteria and standards through checking and critiquing. Creating: Putting elements together to form a novel, coherent whole or an original product; reorganizing elements into a new pattern or structure through generating, planning, or producing (Mercan, 2019, pp. 292–293).

Operating the mental process in accordance with the aforementioned cognitive domain stages when encountering a new learning situation or a problem serves as an indicator that both higher order cognitive skills are utilized and the objectives of constructivist education are achieved. Constructivism is fundamentally based on the shaping of knowledge as a result of an individual’s experiences, observations, and logical analyses (Akınoğlu, 2012). From this perspective, the successful transition between cognitive levels is not merely a linear progression but an active process of “internalizing” and “reconstructing” information. Bloom’s Taxonomy, on the other hand, includes the stages directed toward this shaping process. When learners fail to transcend the initial stages of the taxonomy, it suggests a disruption in the constructivist learning cycle, where theoretical information remains dormant rather than becoming a functional tool for problem-solving. These challenges can be examined through the complementary perspectives of Shulman’s Pedagogical Content Knowledge (PCK), the Revised Bloom’s Taxonomy, and the concept of inert knowledge. Together, these frameworks provide a useful lens for understanding how theoretical pedagogical knowledge is interpreted, transferred, and applied in unfamiliar educational contexts. Within teacher education, a critical gap often emerges between possessing theoretical educational knowledge and executing pedagogical reasoning in professional practice. When pre-service teachers can recall contemporary educational concepts but experience difficulty interpreting and applying them within culturally embedded and unfamiliar contexts, challenges may emerge in transferring theoretical knowledge to pedagogical reasoning. Rather than indicating that cognition remains at a specific cognitive level, such difficulties may suggest limitations in the flexible interpretation and application of pedagogical knowledge across contexts. Consequently, when theoretical definitions are not readily operationalized within novel situations, the resulting difficulties may display characteristics commonly associated with inert knowledge, a phenomenon in which previously acquired knowledge is not easily transferred to new contexts. Bridging this gap requires high-level PCK, which does not merely entail the acquisition of isolated pedagogical definitions, but demands the cognitive fluency to map, decode, and structurally translate abstract educational wisdom into active pedagogical reasoning tools.

The significance of this study lies in its dual contribution to both cultural heritage preservation and contemporary teacher education. Firstly, while Kutadgu Bilig is widely recognized as a literary and moral masterpiece, its potential as a functional tool for developing Pedagogical Content Knowledge (PCK) has remained largely under-researched. By bridging an 11th century classic with the Revised Bloom’s Taxonomy, this research offers a novel interdisciplinary framework that revitalizes ancient wisdom for modern instructional use. Secondly, the study addresses a critical gap in teacher training: the transition from ‘knowing’ to ‘interpreting.’ In an era of high-stakes examinations like the AGS, this research provides an opportunity to examine challenges that may be associated with inert knowledge where candidates possess theoretical information but struggle to apply it to complex educational metaphors. Consequently, the findings may contribute to ongoing discussions regarding the limitations of rote-learning orientations in teacher education and underscore the potential value of culturally sustaining pedagogies (Paris and Alim, 2017).

“In their interpersonal relationships, individuals carry out their face-to-face communication largely through language. Individuals’ competence in using language manifests itself through their ability to establish communication. This situation, in the background, is related to whether the individual can use basic language skills effectively and efficiently. Individuals’ ability to comprehend what they listen to and to express themselves by generating new ideas from what they read and hear is inherently linked to their language proficiency” (Göçer, 2019, p. 51). Extending this logic, the interpretative analysis of classical texts involves more than linguistic decoding; it requires a form of “pedagogical literacy” where the reader identifies deeper structural meanings. Consequently, reading a work like Kutadgu Bilig which conveys numerous religious, political, moral, social, and educational messages to its target audience while also serving as a linguistic and literary treasure in terms of reflecting the linguistic characteristics of the period it was written (Mert, 2009; Doğan, 2010; Türer, 2011; Alyılmaz, 2015) and being able to generate ideas regarding its educational content requires the use of higher order cognitive skills. Being able to bridge the gap between 11th century metaphors and contemporary educational concepts is a sophisticated cognitive task that tests the limits of “knowledge transfer”. Extensive literature exists regarding Kutadgu Bilig, yet existing studies predominantly confine the text to historical or moral-educational analyses. While previous scholarship has extensively documented the historical, linguistic, and moral-educational dimensions of Kutadgu Bilig, existing studies predominantly treat the text as a static object of cultural heritage rather than an active instrument for cognitive evaluation. A significant research gap persists regarding how classical literary metaphors can be operationally utilized to diagnose contemporary teacher competencies. No prior research has instrumentally positioned this 11th century masterpiece as a dynamic pedagogical benchmark or diagnostic tool to evaluate the active pedagogical content knowledge (PCK) and cognitive mapping skills of pre-service teachers, particularly within contemporary teacher education contexts. This study addresses this specific gap by positioning the classical text as a dynamic benchmark for pedagogical reasoning. Based on this premise, this research aims to evaluate the cognitive skills of pre-service Turkish language teachers in terms of their ability to identify educational elements in Kutadgu Bilig. To achieve this aim, the following research questions were addressed:

- To what extent can pre-service Turkish language teachers associate the selected couplets in Kutadgu Bilig with contemporary educational concepts?
- How can the associations established between the selected couplets and pedagogical concepts, together with the participants’ justifications for these associations, be interpreted within the framework of the Revised Bloom’s Taxonomy?
- What do pre-service Turkish language teachers’ interpretations and conceptual associations of the selected couplets reveal about their capacity to interpret and transfer pedagogical knowledge?

## 2 Methodology

This section of the research provides detailed information regarding the research design, the study group, and the processes of data collection and analysis.

### 2.1 Research model

This study, designed qualitatively, is a case study. In case studies, researchers collect detailed and in-depth information about a real-life situation or event and examine a social group or interconnected systems in depth (Lewis, 2003; Yeşilbaş Özenç, 2022). Beyond these foundational definitions, this design was specifically chosen to capture the “bounded” nature of the pedagogical development of pre-service teachers within a specific academic environment (Creswell and Poth, 2024). According to Yin (2018), case studies are instrumental when the boundaries between the phenomenon (pedagogical interpretation) and the context (the educational heritage of Kutadgu Bilig) are not clearly evident. In this study, the case study approach facilitates an “intrinsic” exploration (Stake, 1995) of how teacher candidates engage in the “sense-making” process when mapping historical metaphors onto modern educational frameworks.

### 2.2 Participants

The study group consists of 20 pre-service Turkish language teachers studying at Kafkas University during the 2025–2026 academic year, selected via the convenience sampling method. The primary criteria in this sampling method are ease of access and proximity (Yıldırım and Şimşek, 2008). While convenience sampling is often utilized for its practical advantages, in this study, it was strategically employed to access a specific “information-rich” group (Creswell and Poth, 2024). To ensure this information richness, specific inclusion criteria were applied: all participants had successfully completed core pedagogical courses (including Educational Psychology, Principles and Methods of Teaching, and Measurement and Evaluation) and advanced content knowledge courses (such as Classical Turkish Literature and History of Turkish Education). All of the pre-service Turkish language teachers participating in the study are fourth year (senior) students.

In qualitative case study research, the primary aim is not statistical generalization but analytical depth. Accordingly, the sample size of 20 participants was considered sufficient to enable information-rich analysis and to reach analytical saturation within a bounded educational context. Moreover, the relative homogeneity of the participant group—senior pre-service teachers preparing for a national high-stakes examination—enhances the internal coherence and interpretive validity of the findings.

The main rationale for working with fourth-year students is the assumption that they are in the intensive preparation process for the Ministry of National Education Academy Entrance Examination (AGS). Therefore, they are expected to possess a robust and current knowledge base in educational sciences. This selection aligns with the principles of purposive elements within convenience sampling, as the participants represent a specific developmental stage in their professional journey where theoretical knowledge is at its peak (Saldana, 2021). The personal characteristics of the study group are presented in Table 1.

**Table 1:** Demographic information of the participants.

	*f*	%
Gender		
Female	7	35
Male	13	65
Total	20	100
AGS preparation		
Yes	18	90
No	2	10
Total	20	100
Prior reading of Kutadgu Bilig		
Yes	10	50
No	10	50
Total	20	100

According to Tables 1, 2 of the participants are female and 13 are male. While 18 of the pre-service Turkish language teachers in the study group stated that they were preparing for the AGS, half of the participants expressed that they had previously read Kutadgu Bilig.

**Table 2:** Participants’ responses to couplets related to behaviorism and john locke’s philosophy.

Couplet	Original couplet	Modern Turkish equivalent	Educational equivalent	Participant response		
					*f*	%
1,680	Biligsiz tuğar ol turu ögrenür Bilig bilse ötrü kamuğ iş unar	A person is born without knowledge and learns through experience; once they possess knowledge, they succeed in all their endeavors.	Behaviorism and John Locke’s Philosophy	Correct	8	40
				Incorrect	10	50
				Empty	2	10
				Total	20	100
1,681	Anadın toğuğlı biligsiz tuğar Bilig ögrenür ötrü törke ağar	One who is born of a mother is born without knowledge; they acquire knowledge and thereby gain prestige.	Behaviorism and John Locke’s Philosophy	Correct	8	40
				Incorrect	12	60
				Empty	–	–
				Total	20	100

### 2.3 Data collection and analysis

A form developed by the researchers was used to collect data in the study. The form consists of two sections; the first section includes questions regarding the personal characteristics of the participants. In the second section of the form, 15 couplets selected from Kutadgu Bilig were presented together with their modern Turkish equivalents. The selection of these couplets was based on the pedagogical classifications proposed by Bozkırlı (2015), who identified a range of educational concepts embedded within the text. Participants were asked to associate each couplet with a contemporary educational concept and to justify their associations. These pedagogical classifications were employed as an analytical reference framework rather than as the sole possible interpretations of the couplets, acknowledging the inherently interpretive nature of literary texts. By providing both the original forms and the modern Turkish equivalents of the selected couplets, the pre-service Turkish language teachers were asked to interpret these couplets and associate them with an educational concept. This dual-presentation (original and modern) was designed to minimize linguistic barriers and focus specifically on the participants’ cognitive-pedagogical mapping abilities.

The qualitative data obtained from the participants’ engagement with these couplets were analyzed using deductive thematic analysis. In this approach, the primary operation is to group similar data within the framework of pre-determined concepts and themes, organizing them in an interpretable manner (Yıldırım and Şimşek, 2008). In this context, the selected 15 couplets were initially coded based on their semantic content to identify their target pedagogical counterparts. To ensure institutional objectivity, this initial coding process was conducted collaboratively by an educational science expert and a Turkish language education specialist. Following the completion of the coding phase, the Lawshe (1975) technique was systematically utilized to compute Content Validity Ratios (CVR) and verify the structural appropriateness of the identified pedagogical counterparts in relation to the couplets’ historical and educational content.

This technique, frequently employed to operationalize qualitative expert judgments into quantifiable validity metrics (Yurdugül, 2005; Yurdugül and Bayrak, 2012), was executed through its six established methodological stages: (a) establishing the expert panel, (b) preparing the candidate scale forms, (c) obtaining expert opinions, (d) calculating the content validity ratios for each item, (e) determining the overall content validity index, and (f) final form generation based on CVR/CVI thresholds. Following Lawshe’s criteria, the experts independently evaluated each couplet’s pedagogical mapping using a three-point categorical scale (‘Appropriate/Essential’, ‘Needs Revision/Useful’, and ‘Inappropriate/Not Necessary’). Calculations were subsequently processed based on the number of experts indicating ‘Appropriate/Essential’ to determine critical consensus thresholds (Ayre and Scally, 2014; Lawshe, 1975). The standard formula utilized for this calculation is as follows (Lawshe, 1975; Yurdugül and Bayrak, 2012):


CVR=[Ng−(N/2)]/(N/2)

Where CVR represents the content validity ratio of the item, Ng indicates the number of experts stating ‘Appropriate/Essential’, and N represents the total number of experts. Within the scope of this study, an expert panel consisting of 11 specialists was established to evaluate the structural validity of the pedagogical mappings. The panel comprised one Turkish language teacher with a master’s degree, three research assistants with master’s degrees and one with a PhD in Turkish education, three faculty members in the same field, as well as two research assistants with master’s degrees and one with a PhD in educational sciences. According to Lawshe’s (1975) significance thresholds for a panel of 11 experts, the minimum acceptable CVR value was determined as 0.59 (*p* = 0.05). Items that met or exceeded the 0.59 threshold were retained, thereby mathematically securing the content validity of the instrument.

To further secure the trustworthiness and inter-coder reliability of the subsequent thematic analysis, all participant responses were independently re-analyzed by a co-researcher specializing in both Folk Literature and Turkish Language and Literature Education, who acted as an independent coder. This co-researcher’s dual expertise ensured that the semantic nuances of the 11th century oral and literary traditions were accurately decoded while maintaining strict alignment with contemporary pedagogical training principles. The deductive thematic analysis approach was structured around the pre-established categories of Bozkırlı (2015), which effectively functioned as an analytical “codebook” for evaluating and stabilizing the participants’ responses (Braun and Clarke, 2022; Vaismoradi et al., 2013). In addition, the pedagogical classifications proposed by Bozkırlı (2015) were employed as an analytical reference framework rather than as the sole valid interpretations of the selected couplets. Given the inherently interpretive nature of literary texts, alternative pedagogical readings may also be possible. Within this context, the Revised Bloom’s Taxonomy was not used as a rigid scoring framework but as an interpretive lens for examining the cognitive characteristics reflected in participants’ conceptual associations and written justifications. Accordingly, the taxonomy was employed to explore the extent to which participants moved beyond concept recognition toward interpretation and pedagogical meaning-making. The inter-coder agreement framework recommended by Miles and Huberman (1994) was utilized to compute the consensus level between the researchers. The formula [Agreement/(Agreement + Disagreement) x 100] yielded a reliability coefficient of 0.92. This high degree of interpretive consistency confirmed that researcher bias was effectively minimized and the analysis focused accurately on the semantic and contextual level of meaning (Nowell et al., 2017), establishing a rigorous and transparent methodological foundation before finalizing the findings.

In reporting participant quotations, numerical identifiers (e.g., P1–P20) were assigned to ensure anonymity and confidentiality. These identifiers were used solely for illustrative purposes when presenting representative excerpts from participant responses.

## 3 Findings

This section of the study presents the data obtained from the participants through various tables.

According to Table 3, while the participants were able to identify the correct educational equivalent for couplet 293 related to the concept of maturation at a high rate, they did not demonstrate the same level of success in identifying the correct educational equivalent for couplet 1,050, despite its very clear expression in modern Turkish.

**Table 3:** Participants’ responses to couplets related to maturation.

Couplet	Original couplet	Modern Turkish equivalent	Educational equivalent	Participant response		
					*f*	%
293	Kiçig oğlanığ kör ukuşka ulam Yaşı yetmeginçe yorımaz kılâm	Look at a young boy, wisdom will reach him; but the pens do not move until he comes of age.	Maturation	Correct	14	70
				Incorrect	6	30
				Empty	–	–
				Total	20	100
1,050	Ne kim işler erse tükelin küder Tükegli tükese énişke yanar	Everything awaits its own maturity; once it reaches full maturity, it begins to decline again.	Maturation	Correct	8	40
				Incorrect	11	55
				Empty	1	5
				Total	20	100

In relation to Couplet 293, one participant associated the couplet with the concept of maturation and stated:

“The couplet clearly suggests that a child cannot acquire skills related to science/knowledge before attaining an appropriate level of biological and cognitive maturation” (P15).

This response was coded as correct because the participant accurately identified the developmental process emphasized in the couplet and successfully associated it with the concept of maturation. The explanation reflects an understanding that certain abilities emerge only after an individual reaches the necessary level of biological and cognitive development.

According to Table 4, while the participants had a very low success rate in identifying the correct equivalent for couplet 636, which is associated with the concepts of reward and reinforcement, they achieved a high success rate in identifying the correct equivalent for couplet 1,494, related to the concepts of punishment and reinforcement.

**Table 4:** Participants’ responses to couplets related to reward, punishment, and reinforcement.

Couplet	Original couplet	Modern Turkish equivalent	Educational equivalent	Participant response		
					*f*	%
636	Bışurğu tapuğda sınağu körü Ağırlasa ötrü kötürgü örü	A servant should first be matured through service and thoroughly tested; only then should they be promoted and rewarded.	Reward, Reinforcement	Correct	5	25
				Incorrect	15	75
				Empty	-	-
				Total	20	100
1,494	Ayama oğul kızka berge yétür Oğul kızka berge bilig ögretür	If necessary, beat the son and the daughter without pity; corporal punishment teaches knowledge to children.	Punishment and Reinforcement	Correct	14	70
				Incorrect	5	25
				Empty	1	5
				Total	20	100

Regarding Couplet 1,494, one participant associated the couplet with the concept of punishment and stated:

“Every behavior, whether positive or negative, has a consequence. Individuals should be aware of these consequences and regulate their behavior accordingly” (P7).

This response was coded as correct because the participant recognized the relationship between behavior and its consequences. The explanation demonstrates an understanding that individuals may regulate their actions based on the outcomes of their behavior, which is consistent with the concept of punishment emphasized in the couplet.

As shown in Table 5, participants demonstrated an equal success rate in identifying the educational counterparts for couplets 881 and 883 regarding the concept of heredity. However, less than half of the Turkish teacher candidates successfully linked these couplets to hereditary factors.

**Table 5:** Participants’ responses to couplets related to heredity.

Couplet	Original couplet	Modern Turkish equivalent	Educational equivalent	Participant response		
					*f*	%
881	Ürüñ süt bile kirse edgü kılık Ölüm tutmağınça ewürmez yorık	If goodness enters one’s soul through the mother’s pure milk, that person will not stray from the righteous path until death.	Heredity	Correct	8	40
				Incorrect	11	55
				Empty	1	5
				Total	20	100
883	Karında törümiş kılınç ögretig Yağız yér katında kiter ay tetig	The nature and nurture formed in the mother’s womb leave a person only under the black earth, O wise one.	Heredity	Correct	8	40
				Incorrect	11	55
				Empty	1	5
				Total	20	100

Regarding Couplet 883, one participant associated the couplet with the concept of heredity and stated:

“Based on the expression ‘in the mother’s womb’ in this couplet, I thought that genetic factors are among the elements that shape human personality, and I therefore reached this conclusion” (P11).

This response was coded as correct because the participant identified the reference to characteristics acquired before birth and interpreted the couplet in relation to hereditary influences on human development. The explanation demonstrates an understanding that certain individual characteristics may originate from factors associated with heredity.

Conversely, an in-depth thematic analysis of the incorrect associations within Table 5 reveals that the pre-service teachers’ misinterpretations were not arbitrary or random guesses; instead, they emerged from alternative pedagogical frameworks that carried an underlying educational logic. For instance, regarding the 881st couplet, which inherently highlights the unyielding nature of hereditary traits, one pre-service teacher (P17) categorized the couplet under the theme of “Values Education,” providing the following written rationale:

“I understood from this couplet that values are beneficial to the person and become behavior over time” (P17).

From the perspective of Shulman’s Pedagogical Content Knowledge (PCK) framework, this incorrect association demonstrates that while the participant failed to decipher the biological and developmental constraints implied by the historical text (heredity), they actively engaged in a constructive meaning-making process. The participant linked the concept of lifelong “permanence” expressed in the couplet to the internalization of moral values and habit formation, interpreting the structural stability of human nature through a contemporary pedagogical lens. This pattern suggests that when pre-service teachers encountered historical metaphors that triggered cognitive conflict, they tended to rely heavily on modern, highly familiar educational discourses—such as character development and values education—to bridge their conceptual gaps when they could not fully map the underlying developmental philosophy of the text.

According to Table 6, the participants identified the correct equivalents for couplets 884 and 1,297, which are associated with the concepts of modeling and social learning, at a very low rate. Only a small fraction of the pre-service Turkish teachers were able to associate these couplets with the concepts of modeling and social learning.

**Table 6:** Participants’ responses to couplets related to modeling and social learning.

Couplet	Original couplet	Modern Turkish equivalent	Educational equivalent	Participant response		
					*f*	%
884	Kalı edgüke bolsa isiz işi Isiz boldı kılkı ol isiz tuşı	If a good person gains a bad companion, their nature will also turn bad, just like their companion’s.	Modeling and social learning	Correct	4	20
				Incorrect	16	80
				Empty	–	–
				Total	20	100
1,297	Isiz işke yakma saña kılğa kor Isizlik yılan ol séni tikge kör	Do not approach a bad companion, for they will bring you harm; evil is a serpent, beware, it will sting you.	Modeling and social learning	Correct	3	15
				Incorrect	15	75
				Empty	2	10
				Total	20	100

Regarding Couplet 884, one participant associated the couplet with the concept of reverse effect and stated:

“The couplet suggests that when good and bad come together, it is not goodness but rather evil that becomes dominant” (P1).

Although this response was coded as incorrect according to the analytical framework adopted in the study, it reflects a meaningful attempt to interpret the message of the couplet. The participant recognized the influence of interpersonal interaction on behavior; however, rather than associating the couplet with modeling and social learning, the interpretation focused on the predominance of negative influence within social relationships.

As shown in Table 7, while participants demonstrated a higher success rate in linking couplet 1,493 to the critical period than they did for couplet 1,495, the overall association rate for the former remained at only 50 %.

**Table 7:** Participants’ responses to couplets related to the critical period.

Couplet	Original couplet	Modern Turkish equivalent	Educational equivalent	Participant response		
					*f*	%
1,493	Kiçig erken ögret oğulka bilig Kiçigde bilig bilse kötrür elig	Teach the son knowledge while he is young; if he learns in his youth, he will be successful in life.	Critical Period	Correct	10	50
				Incorrect	10	50
				Empty	-	-
				Total	20	100
1,495	Kiçiglikte bilse oğul kız neni Karıp ölmeginçe unıtmaz anı	Whatever a son or daughter learns in childhood, they will not forget until they grow old and die.	Critical Period	Correct	6	30
				Incorrect	14	70
				Empty	-	-
				Total	20	100

Regarding Couplet 1,493, one participant associated the couplet with the concept of preschool education rather than the concept of the critical period and provided the following explanation:

“The couplet emphasizes that education should begin at an early age. By saying ‘teach while young,’ it suggests that learning should start before formal schooling and that parents or adults have a responsibility to provide guidance during this period” (P8).

Although this response was coded as incorrect according to the analytical framework adopted in the study, it demonstrates a meaningful interpretation of the educational message conveyed in the couplet. The participant recognized the importance of early childhood education and the role of adults in the learning process. While the concept of the critical period was not explicitly identified, the response reflects an understanding closely related to the significance of early developmental stages for learning.

As illustrated in Table 2, participants demonstrated a poor success rate in linking couplets 1,680 and 1,681 to the frameworks of behaviorism and Lockean philosophy. Less than 50 % of the pre-service teachers successfully correlated these couplets with the aforementioned conceptual categories.

Regarding Couplet 1,680, one participant associated the couplet with the concept of maturation and stated:

“Human beings are born without knowledge. As they gain experience, they learn; as they learn, they become more competent; and as they become more competent, they gain respect. Therefore, competence is important” (P5).

Although this response was coded as incorrect according to the analytical framework adopted in the study, it reflects a meaningful interpretation of the developmental process described in the couplet. The participant recognized the relationship between learning, personal growth, and competence; however, rather than associating the couplet with the idea that knowledge is acquired through experience and environmental influence, the interpretation focused primarily on maturation and individual development.

According to Table 8, the participants demonstrated a very low rate of accuracy in identifying the correct equivalents for couplets 4,380, 4,381, and 4,382, which are related to the simple-to-complex learning principle. Only two of the participants were able to associate these couplets with the principle of simple-to-complex learning.

**Table 8:** Participants’ responses to couplets related to the principle of simple-to-complex learning.

Couplet	Original couplet	Modern Turkish equivalent	Educational equivalent	Participant response		
					*f*	%
4,380	Ya taz’îf ya tansîf özüñ yetrü bil Kalı bildiñ erse ‘aded cedri kıl	Master the doubling and halving [of numbers] thoroughly; only after learning these should you take up the square root.	The Principle of Simple-to-Complex	Correct	2	10
				Incorrect	11	55
				Empty	7	35
				Total	20	100
4,381	Yana cem’ ü tefrîk misâhatka öt Yéti kat felekni yatur yamça tut	Then proceed to addition, subtraction, and measurement; [thus] hold the seven layers of the heavens in your palm as if they were a mere splinter.	The Principle of Simple-to-Complex	Correct	2	10
				Incorrect	11	55
				Empty	7	35
				Total	20	100
4,382	Takı kolsa cebr ü mukâbel okı Yéme oklidis kapğı yetrü tokı	If you desire even more, study algebra and equations; furthermore, knock thoroughly on the door of Euclid.	The Principle of Simple-to-Complex	Correct	2	10
				Incorrect	11	55
				Empty	7	35
				Total	20	100

Regarding Couplet 4,380, one participant left the item unanswered and provided the following explanation:

“This couplet did not evoke any particular idea for me. I could not understand it” (P16).

Another participant also left the item unanswered and stated:

“I do not think that this couplet has any educational value” (P3).

These responses suggest that some participants experienced substantial difficulty in interpreting the educational meaning embedded in the couplet. While one participant was unable to construct any meaningful interpretation, another did not perceive the couplet as conveying an educational message at all. Taken together, these responses indicate that certain couplets posed considerable challenges for participants in establishing connections between classical literary expressions and contemporary pedagogical concepts.

When interpreted through the framework of the Revised Bloom’s Taxonomy, the findings suggest that although participants were generally able to associate the selected couplets with educational concepts, they experienced varying degrees of difficulty in constructing conceptually accurate and pedagogically grounded interpretations. The responses indicate challenges in moving beyond concept recognition toward deeper levels of interpretation and pedagogical meaning-making. These findings may reflect limitations in the flexible application and transfer of pedagogical knowledge to culturally embedded and unfamiliar contexts.

## 4 Discussion

The present study examined how pre-service Turkish language teachers associated selected couplets from Kutadgu Bilig with contemporary educational concepts and how these associations reflected their pedagogical interpretation skills. The findings revealed that all participants were able to establish some form of relationship between the selected couplets and educational concepts. This finding supports previous studies emphasizing the educational richness of Kutadgu Bilig and its potential as a source of pedagogical insight (Akyüz, 2002; Özkan, 2011; Akyüz, 2013; Bozkırlı, 2015; Kaya, 2017; Koçak and Dönmez, 2018; Şimşek, 2021). Participants were generally able to identify educational themes within the selected couplets, suggesting that the pedagogical messages embedded in Kutadgu Bilig remain accessible and meaningful for contemporary readers.

However, the findings also indicated that participants experienced varying degrees of difficulty in establishing conceptually accurate associations. Although educational concepts were frequently identified, the majority of responses did not fully correspond with the pedagogical framework adopted in this study. This finding suggests that recognizing an educational theme and accurately interpreting its pedagogical significance may involve different levels of cognitive engagement. As Shulman (1987) argues, pedagogical content knowledge requires more than familiarity with educational terminology; it also involves the ability to interpret, contextualize, and apply knowledge within meaningful educational situations.

When considered within the framework of the Revised Bloom’s Taxonomy, the findings suggest that many participants encountered challenges in moving beyond concept recognition toward more elaborate forms of interpretation and pedagogical meaning-making. Rather than indicating an absence of higher-order thinking, the findings point to difficulties in constructing conceptually precise and pedagogically grounded interpretations. In this respect, the study highlights the complexity of transferring theoretical pedagogical knowledge to culturally embedded and unfamiliar contexts.

The findings may also be interpreted in relation to the concept of inert knowledge. According to Renkl et al. (1996), inert knowledge refers to knowledge that can be recalled but is not readily transferred to new situations or applied flexibly in unfamiliar contexts. Although the present study does not directly measure inert knowledge, the observed difficulties in establishing pedagogically accurate interpretations appear consistent with characteristics commonly associated with this phenomenon. Therefore, the findings should be viewed not as direct evidence of inert knowledge but as an indication that some participants may experience challenges in applying previously acquired pedagogical knowledge in interpretive tasks.

Another noteworthy finding concerns the educational potential of culturally grounded texts in teacher education. The results demonstrate that classical literary works such as Kutadgu Bilig can serve not only as cultural heritage texts but also as valuable pedagogical resources that encourage interpretation, conceptual reasoning, and knowledge transfer. In this regard, the study supports the argument of Paris and Alim (2017) that culturally sustaining pedagogies should create opportunities for learners to engage actively with cultural knowledge rather than merely reproduce it.

Taken together, these findings point to the importance of creating learning environments that encourage interpretation, contextualization, and pedagogical reasoning alongside the acquisition of theoretical knowledge. Developing such competencies may help pre-service teachers transform pedagogical concepts from isolated theoretical constructs into flexible tools for educational interpretation and practice.

## 5 Conclusion

This study examined how pre-service Turkish language teachers associated selected couplets from Kutadgu Bilig with contemporary educational concepts and interpreted the pedagogical meanings embedded within these classical texts. The findings indicate that although participants were generally able to identify educational themes, they experienced varying degrees of difficulty in constructing conceptually accurate and pedagogically grounded interpretations.

The study contributes to the literature by demonstrating that Kutadgu Bilig can function not only as a literary and cultural work but also as a pedagogical resource for exploring cognitive-pedagogical engagement in teacher education. The findings further suggest the importance of supporting pre-service teachers in developing interpretive competence and pedagogical reasoning alongside theoretical knowledge acquisition.

Overall, the study highlights the potential value of integrating culturally rooted texts into teacher education programs as a means of encouraging deeper pedagogical interpretation, contextualization, and knowledge transfer.

## 6 Limitations

This study has several limitations that should be considered when interpreting the findings. First, the study was conducted with a relatively small group of pre-service Turkish language teachers from a single institution, which limits the transferability of the findings. Second, the analysis was based on participants’ responses to a specific pedagogical mapping task and therefore reflects performance within a particular context. Finally, although the pedagogical classifications proposed by Bozkırlı (2015) were used as an analytical reference framework, alternative pedagogical interpretations of literary texts may also be possible.

## 7 Implications and future research

The findings suggest that teacher education programs may benefit from incorporating activities that require the interpretation of culturally embedded educational messages and the transfer of pedagogical knowledge across contexts. Such activities may support the development of pedagogical reasoning and interpretive competence.

Future studies may involve larger and more diverse participant groups, compare different teacher education programs, and employ interviews or think-aloud protocols to gain deeper insight into participants’ cognitive and pedagogical reasoning processes.

## Data Availability

The raw data supporting the conclusions of this article will be made available by the authors, without undue reservation.
