# Supplementary material for: Bridging classical wisdom and modern pedagogy: pre-service teachers’ cognitive engagement with Kutadgu Bilig
Source: Front Psychol. 2026 Jul 1;17:1878026. doi: 10.3389/fpsyg.2026.1878026 (PMC13368928; doi:10.3389/fpsyg.2026.1878026)
Supplement: Supplementary file 1 [file Supplementary_file_1.pdf]

Appendix A. English Translation of the Data Collection Instrument

PEDAGOGICAL MAPPING FORM FOR SELECTED KUTADGU BILIG COUPLETS

Informed Consent Statement:

This study is a scientific research project conducted to examine the potential use of classical literary texts as pedagogical tools in teacher education and to evaluate pre-service teachers' cognitive association processes. The data collected through this form will be used solely for research and academic publication purposes (e.g., journal articles, conference papers), and all personal information will remain strictly confidential. Participation in this study is entirely voluntary. Participants may discontinue completing the form at any time without any obligation. By completing this form, participants consent to the anonymous processing and use of their responses for scientific purposes. Thank you for your contribution.

[ ] I have read the statement above and voluntarily agree to participate in this study. I consent to the anonymous use of my responses for academic and scientific purposes.

Instructions:

The table below contains selected couplets from Kutadgu Bilig. For each couplet, please identify the pedagogical concept or principle from contemporary educational sciences (Developmental Psychology, Educational Psychology, or Principles and Methods of Teaching) that you consider most appropriate. In addition, provide a justification explaining the reasoning behind your association in the relevant column.

Personal Information:

1. Gender [ ] Female [ ] Male

2. Have you previously read Kutadgu Bilig? [ ] Yes [ ] No

3. Are you currently preparing for the Academy Entrance Examination (AGS)? [ ] Yes [ ] No

Couplet	Original Couplet	English Translation	Educational Concept
**293**	kiçig oğlanığ kör ukuşka ulam<br>yaşı yetmegince yorımaz kılâm	Look at a young boy, wisdom will reach him; but the pens do not move until he comes of age.	
	**Please explain the reasoning behind your association:**		
**636**	bışurğu tapuğda sınağu körü<br>ağırlasa ötrü kötürgü örü	A servant should first be matured through service and thoroughly tested; only then should they be promoted and rewarded.	
	**Please explain the reasoning behind your association:**		
**881**	ürüñ süt bile kirse edgü kılık<br>ölüm tutmağınça ewürmez yorık	If goodness enters one's soul through the mother's pure milk, that person will not stray from the righteous path until death.	
	**Please explain the reasoning behind your association:**		
**883**	karında törümiş kılınç ögretig<br>yağız yér katında kiter ay tetig	The nature and nurture formed in the mother's womb leave a person only under the black earth, O wise one.	
	**Please explain the reasoning behind your association:**		

Appendix B. Sample Participant Response

KUTADGU BİLİG BEYİTLERİNİN EĞİTSEL KARŞILIKLARINI BELİRLEME FORMU

Bilimsel Araştırma Gönüllü Onam Metni:

Bu çalışma; klasik edebî metinlerin öğretmen eğitiminde pedagojik bir araç olarak kullanılabilirliğini ve öğretmen adaylarının bilişsel ilişkilendirme süreçlerini değerlendirmek amacıyla yürütülen bilimsel bir araştırmadır. Bu formu doldurarak sağlayacağınız veriler, kimlik bilgileriniz tamamen gizli tutulmak kaydıyla, yalnızca akademik ve bilimsel yayınlarda (makale, bildiri vb.) veri analizi amacıyla kullanılacaktır. Araştırmaya katılımınız tamamen gönüllülük esasına dayalıdır; istediğiniz an formu doldurmayı bırakabilirsiniz. Formu doldurarak verilerinizin bilimsel amaçlarla anonim olarak işlenmesini kabul etmiş sayılmaktasınız. Katkılarınız için teşekkür ederiz.

[✓] **Yukarıdaki metni okudum, çalışmaya gönüllü olarak katılmayı ve verilerimin anonim olarak akademik yayınlarda kullanılmasını onaylıyorum.**

Yönerge:

Aşağıdaki tabloda, Kutadgu Bilig'den seçilmiş örnek beyitler yer almaktadır. Her beyit için çağdaş eğitim bilimleri müfredatından (Gelişim Psikolojisi, Öğrenme Psikolojisi veya Öğretim İlke ve Yöntemleri) en uygun bulduğunuz **pedagojik kavramı/ilkeyi** yazınız ve bu bağlantıyı kurarken kullandığınız **gerekçeli açıklamayı (mantıksal akıl yürütmeyi)** ilgili sütunlara yazınız.

Kişisel Bilgiler:

1. Cinsiyet [✓] Kız [ ] Erkek

2. Kutadgu Bilig'i daha önce okudunuz mu? [ ] Evet [✓] Hayır

3. AGS'ye (Akademi Giriş Sınavı) hazırlanıyor musunuz? [✓] Evet [ ] Hayır

Beyit Numarası	Orijinal Beyit	Günümüz Karşılığı	Eğitsel Karşılığı
293	kiçig oğlanığ kör ukuşka ulam<br>yaşı yetmegince yorımaz kılâm	Küçük bir oğlana bak, akıl ona ulaşacaktır; fakat yaşı gelmedikçe kalemler yürümez.	Olgunlaşma
	**Neden bu karşılığı verdiğinizi açıklayınız:** Bir çocuğun biyolojik ve zihinsel olarak belirli bir gelişim seviyesine ulaşmadan belirli becerileri kazanamayacağı açıkça belirtilmektedir.		
636	bışurğu tapuğda sınağu körü<br>ağırlasa ötrü kötürgü örü	Kulu önce hizmette pişirmeli ve iyice sınamalı, ondan sonra kendisini yükseltip ödüllendirmeli.	Yakından uzağa
	**Neden bu karşılığı verdiğinizi açıklayınız:** Bir kişinin hizmette pişirilmesi ve sınanması eğitimde kademeli yaklaşımı ve yaparak yaşayarak öğrenmeyi simgeler.		
881	ürüñ süt bile kirse edgü kılık<br>ölüm tutmağınça ewürmez yorık	Eğer iyilik ananın ak sütü ile insanın ruhuna girerse, o kişi ölünceye kadar doğru yoldan çıkmaz.	Kişilik gelişimi
	**Neden bu karşılığı verdiğinizi açıklayınız:** Yaşamın ilk yerlerinde şekillenen kalıcı izler bırakır.		
883	karında törümiş kılınç ögretig<br>yağız yér katında kiter ay tetig	Ana karnında oluşan doğa ve terbiye ancak kara toprak altında insanı terk eder; ey zeki.	Kalıtım ve Cevre
	**Neden bu karşılığı verdiğinizi açıklayınız:** İnsan gelişiminin kalıtım ve cevrenin ortak ürünüdür.		

Appendix C. Data Collection Process

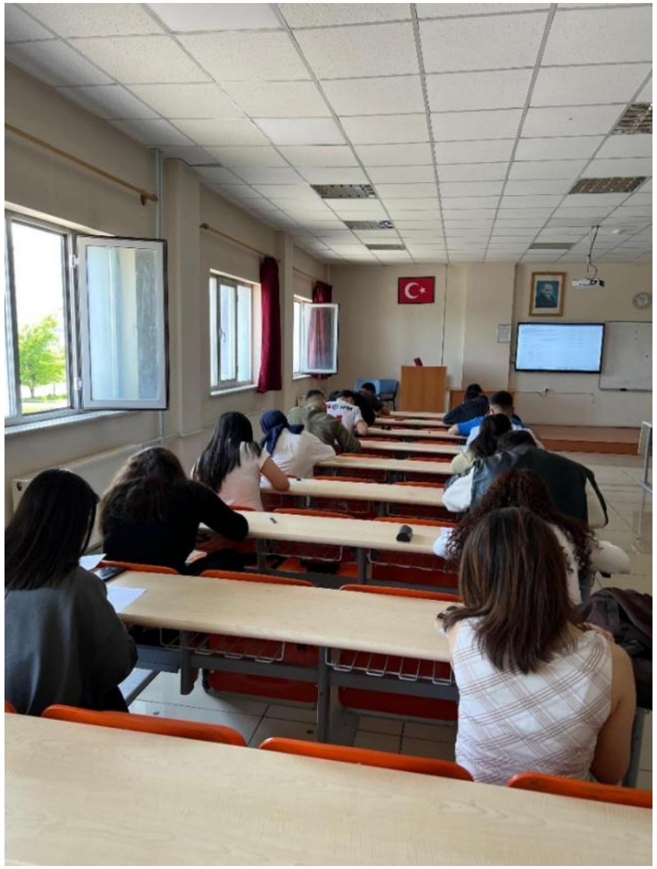

Figure C1. Participants completing the pedagogical mapping task during the data collection process

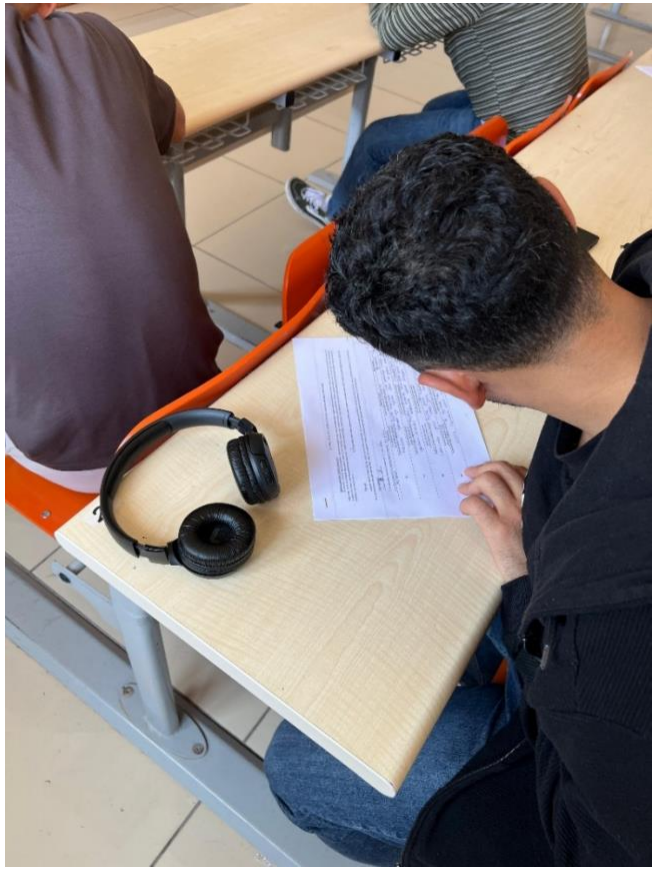

Figure C2. Participants engaging in the pedagogical mapping activity based on selected couplets from Kutadgu Bilig
